# A novel NF-κB inhibitor, DHMEQ, ameliorates pristane-induced lupus in mice

**DOI:** 10.3892/etm.2014.1718

**Published:** 2014-05-19

**Authors:** HUIQING QU, WEIHUA BIAN, YANYAN XU

**Affiliations:** 1 Department of Blood Transfusion, Affiliated Hospital of Binzhou Medical College, Binzhou, Shandong 256603, P.R. China; 2 Department of Biochemistry, Binzhou Medical College, Binzhou, Shandong 256603, P.R. China

**Keywords:** systemic lupus erythematosus, nuclear factor-κB, dehydroxymethylepoxyquinomicin, lupus model, pristane

## Abstract

Nuclear factor (NF)-κB is strongly associated with the development of immune regulation and inflammation. The aim of the present study was to identify whether a NF-κB inhibitor, dehydroxymethylepoxyquinomicin (DHMEQ), ameliorates systemic lupus erythematosus (SLE) in a pristane-induced mouse model. SLE was induced in 8-week-old female BALB/c mice by the injection of 0.5 ml pristane. The therapeutic effect of 12 mg/kg DHMEQ on the pristane-induced BALB/c mouse model of lupus was investigated to elucidate the effects on SLE. The intraperitoneal administration of DHMEQ three times per week was initiated when the mice were 16 weeks-old (8 weeks following the pristane injection) and the treatment was continued for 16 weeks. Serum IgG autoantibodies against nucleosomes, dsDNA and histones were detected at weeks 8, 16 and 32. In addition, the expression levels of interleukin (IL)-1β, 6 and 17, as well as tumor necrosis factor (TNF)-α, were analyzed at week 32. Renal lesions were also observed. DHMEQ was shown to antagonize the increasing levels of anti-nucleosome, anti-dsDNA and anti-histone autoantibodies, as well as the increasing levels of IL-1β, 6 and 17 and TNF-α. In addition, DHMEQ reduced the number of renal lesions caused by pristane, as reflected by milder proteinuria and reduced renal pathology. The renal expression levels of phosphorylated-p38 mitogen-activated protein kinase (MAPK), phosphorylated-c-Jun N-terminal kinase (JNK) and NF-κB p65 were significantly downregulated. Therefore, the results of the present study indicate that DHMEQ has a beneficial effect on pristane-induced lupus through regulating cytokine levels and the MAPK/JNK/NF-κB signaling pathway.

## Introduction

Systemic lupus erythematosus (SLE) is a prototypic autoimmune disease that is characterized by humoral autoimmunity followed by cellular and innate immune responses (1). These responses in turn lead to inflammation and damage to organs, including the kidney, skin and joints. Lupus nephritis (LN) affects the majority of patients with lupus and is associated with poor prognosis in SLE (2). Although the prognosis of patients with SLE has greatly improved, immune suppression remains evident and results in higher susceptibility to infectious and malignant diseases (3). Toxic effects and, in certain cases, unexpectedly severe complications of the current therapies are being increasingly reported (3). Thus, more effective and less toxic therapies for SLE and LN are required.

Nuclear factor (NF)-κB, a key transcription factor involved in the regulation of immune responses, has been demonstrated to be a potential candidate for studies concerning the pathogenesis of autoimmunity (4). Stimulation of a wide range of receptors, including tumor necrosis factor (TNF) receptor, interleukin (IL)-1 receptor, Toll-like receptor, T- and B-cell receptors, B-cell activating factor receptor, receptor activator of NF-κB and CD40, may lead to the activation of NF-κB. Thus, NF-κB functions at the crossroads of a number of signaling pathways (5). Susceptibility genes involved in the NF-κB signaling pathway may synergistically contribute to the increased risk of SLE (6). Thus, in the present study, the potential of a NF-κB inhibitor, dehydroxymethylepoxyquinomicin (DHMEQ), was investigated as a treatment option for LN and SLE using a pristane-induced lupus model.

## Materials and methods

### Animals

Female BALB/c mice (age, 8 weeks; weight, 18±2 g) were supplied by the Experimental Animal Center of Binzhou Medical College (Binzhou, China). All experimental protocols reported in the study were approved by the Ethics Review Committee for Animal Experimentation of Binzhou Medical College.

### Experimental protocols

Female BALB/c mice were randomly divided into vehicle-treated model and DHMEQ-treated groups (n=12 per group). The mice were administered an intraperitoneal injection of 0.5 ml pristane (7). When the mice reached 16-weeks of age (8 weeks following pristane treatment when antibody production was significant), intraperitoneal injections of DHMEQ or vehicle were administered three times a week (8) until the mice were 32 weeks old.

DHMEQ was dissolved in 0.5% carboxymethyl cellulose (CMC) to a final concentration of 1.2 mg/ml, as described previously (8). Intraperitoneal injections of 12 mg/kg DHMEQ or 0.5% CMC (vehicle) were administered. Serum samples were collected from the tail vein at weeks 8, 16 and 32 to measure the level of autoantibodies. At week 32 (24 weeks following pristane administration), the mice were sacrificed under carbon dioxide and nephritic tissues were removed for immunological detection and examination by light microscopy.

### Determination of autoantibodies

Serum levels of IgG autoantibodies against nucleosomes, dsDNA and histones were determined by enzyme-linked immunosorbent assays, as previously described (9,10). Serum IgG autoantibodies against nucleosomes, dsDNA and histones were detected at weeks 8, 16 and 32.

### Determination of cytokines

Expression levels of mouse IL-1β, 6 and 17 and TNF-α were measured using a FlowCytomix bead-based assay (Bender MedSystems, Vienna, Austria), according to the manufacturer’s instructions. Cytokine concentrations were determined using FlowCytomixPro 2.2.0 analysis software (Bender MedSystems).

### Assessment of kidney disease

To assess urinary protein excretion, mice were placed in metabolic cages and urine samples were collected over a 24-h period. Protein concentrations were determined using Coomassie brilliant blue (Pierce Biotechnology, Inc., Rockford, IL, USA), according to the manufacturer’s instructions. Saline-perfused kidney sections obtained from the mice and age-matched C57BL/6 mice were prepared as previously described (11) and analyzed using an Olympus BX60 microscope (Olympus Corporation, Tokyo, Japan). Staining intensity was quantified using Image J software (National Institutes of Health, Bethesda, MD, USA).

### Western blot analysis of kidney proteins

For western blot analysis, the kidneys were removed and snap-frozen in liquid nitrogen for protein extraction using RIPA (Santa Cruz Biotechnology, Inc., Santa Cruz, CA, USA). Next, 20 μg crude protein was subjected to 12.5% sodium dodecyl sulfate-polyacrylamide gel electrophoresis. Following electrophoresis, the proteins were transferred onto a nitrocellulose membrane (GE-Healthcare, Little Chalfont, UK). The blots were blocked with 2% bovine serum albumin in phosphate-buffered saline (PBS), which was followed by 1 h incubation with 1:2,000 diluted primary antibodies against phosphorylated-p38 mitogen-activated protein kinase (p-p38 MAPK), phosphorylated-c-Jun N-terminal kinase (p-JNK) or NF-κB p65 (Santa Cruz Biotechnology, Inc.). Following washing with PBS, the reacted blots were incubated with peroxidase-conjugated secondary antibodies (BioSource International, Inc., Camarillo, CA, USA). Antigen-antibody complexes were identified by enhanced chemiluminescence (Pierce Biotechnology, Inc.) and the photographic density was quantified using an image analysis system (Fujifilm, Tokyo, Japan). GAPDH and lamin B1 were used as internal controls.

### Statistical analysis

Statistical analysis was performed using SPSS software, version 17.0 (SPSS, Inc., Chicago, IL, USA). Data are presented as mean ± SEM. Data were compared by one-way analysis of variance and P<0.01 was considered to indicate a statistically significant difference.

## Results

### DHMEQ reduces the serum levels of anti-dsDNA, anti-nucleosome and anti-histone antibodies in mice with pristane-induced lupus

**Figure 1:**
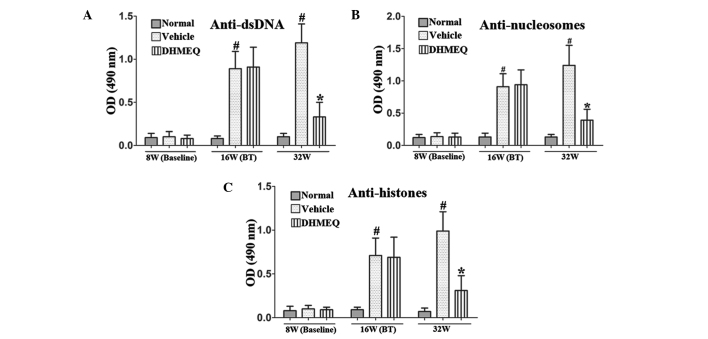
DHMEQ reduced the serum levels of (A) anti-dsDNA, (B) anti-nucleosome and (C) anti-histone autoantibodies in mice with pristane-induced lupus. Serum samples from the mice in each group were collected at weeks 8 (baseline), 16 (prior to treatment) and 32 and the antibody levels were detected by enzyme-linked immunosorbent assays. Optical density (OD) was calculated at 490 nm. Data are presented as mean ± SEM (n=12 per group). ^#^P<0.01, vs. baseline at week 8; ^*^P<0.01, vs. vehicle-treated model control mice. DHMEQ, dehydroxymethylepoxyquinomicin.

The production of numerous autoantibodies is a key feature of SLE and is associated with renal damage. Serum IgG autoantibodies against nucleosomes, dsDNA and histones were detected at weeks 8, 16 and 32. There were marked increases in the serum anti-dsDNA antibody levels of the vehicle-treated model group at weeks 16 and 32, whereas the DHMEQ-treated group had markedly reduced anti-dsDNA antibody levels compared with those in the vehicle-treated model group at week 32 (Fig. 1A). Similarly, serum anti-nucleosome and anti-histone antibody levels were also significantly reduced by DHMEQ treatment when compared with those in the vehicle-treated control group at week 32 (Fig. 1B and C).

### DHMEQ reduces the expression of proinflammatory cytokines in mice with pristane-induced lupus

**Figure 2:**
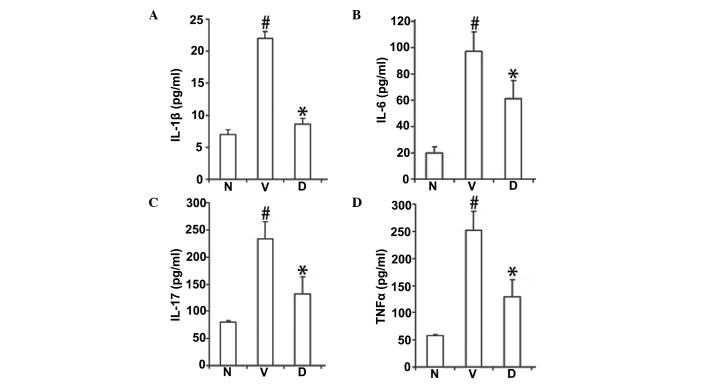
DHMEQ reduced the expression levels of serum (A) IL-1β, (B) IL-6, (C) IL-17 and (D) TNF-α proinflammatory cytokines. Data are expressed as mean ± SEM (n=12 per group). ^#^P<0.01, vs. baseline at week 8; ^*^P<0.01, vs. vehicle-treated model control mice. N, normal; V, vehicle-treated model control; D, DHMEQ-treated model mice; DHMEQ, dehydroxymethylepoxyquinomicin; IL, interleukin; TNF, tumor necrosis factor.

To investigate the effects of DHMEQ on cytokines in SLE, the expression levels of IL-1β, 6 and 17 and TNF-α were analyzed. The results revealed that the expression levels of TNF-α, IL-1β, 6 and 17 were significantly increased in the vehicle-treated SLE model mice (P<0.01; Fig. 2). By contrast, in the DHMEQ treatment group, the cytokine levels were almost normal and markedly lower compared with those in the vehicle-treated model mice (P<0.01; Fig. 2).

### DHMEQ reduces LN in mice with pristane-induced lupus

**Figure 3:**
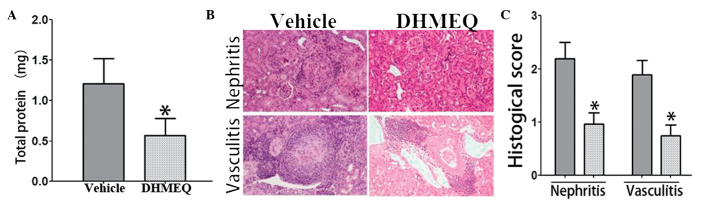
Decreased proteinuria, nephritis and vasculitis was observed in DHMEQ-treated mice. (A) Total urine proteins. (B) Representative histological images of nephritis (upper panels) and vasculitis (lower panels) in 32-week-old mice (hematoxylin and eosin; magnification, ×200). (C) Semiquantitative analysis of nephritis and vasculitis scores. Data are expressed as mean ± SEM. ^*^P<0.01, vs. vehicle-treated model control. LN, lupus nephritis; DHMEQ, dehydroxymethylepoxyquinomicin.

To quantify urinary protein excretion, urine samples from individual mice in metabolic cages were collected over 24 h. At week 32 (24 weeks following pristane injection), urinary protein excretion in the DHMEQ-treated mice was significantly lower compared with that in the vehicle-treated model mice (Fig. 3A).

In addition, the renal histology of the mice with pristane-induced lupus was investigated. Consistent with the aforementioned observations, hematoxylin and eosin-stained sections of the kidneys from mice in the DHMEQ-treated group exhibited reduced glomerular damage compared with that observed in the vehicle-treated lupus model mice (Fig. 3B and C).

### DHMEQ inhibits renal NF-κB and JNK/p38 MAPK pathways in mice with pristane-induced lupus

**Figure 4:**
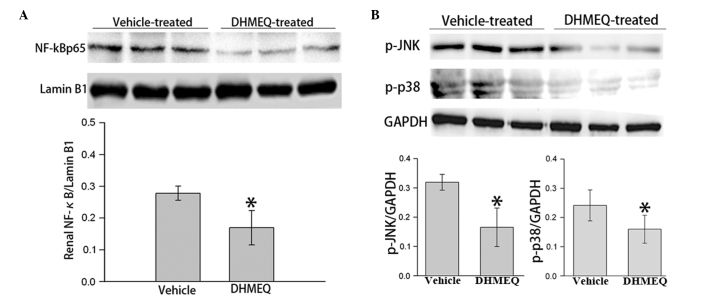
DHMEQ treatment suppressed the activation of the NF-κB and JNK/p38 MAPK signaling pathways. Crude proteins obtained from the kidney tissues of vehicle- and DHMEQ-treated mice were analyzed by western blot analysis using specific antibodies against (A) NF-κB p65 and (B) p-JNK and p-p38 MAPK. Quantitative analysis was performed by densitometry using an internal control. ^*^P<0.01, vs. vehicle-treated control. DHMEQ, dehydroxymethylepoxyquinomicin; NF, nuclear factor; JNK, c-Jun N-terminal kinase; MAPK, mitogen-activated protein kinase; p-JNK, phosphorylated-JNK; p-p38, phosphorylated-p38.

To further explore the underlying mechanism, the expression levels of associated signaling molecules in the renal tissue were analyzed. A previous study indicated that the inflammation-mediated activation of the JNK and p38 MAPK signaling pathways may be the underlying intracellular mechanism causing lymphocyte hyperactivity in SLE (12). NF-κB p65, an indicator of NF-κB signaling activation, was found to be downregulated by DHMEQ treatment (Fig. 4A). In addition, the phosphorylation levels of JNK and p38 MAPK in the renal tissues of the mice with pristane-induced lupus were significantly decreased following DHMEQ treatment (Fig. 4B). These results indicate that the treatment effects of DHMEQ are associated with JNK/p38 MAPK signaling in mouse SLE.

## Discussion

DHMEQ, a 5-dehydroxymethyl derivative of epoxyquinomicin C (13), is an antibiotic originally isolated from *Amycolatopsis* sp. (14). The majority of NF-κB inhibitors target IκBα phosphorylation, whereas DHMEQ inhibits the nuclear translocation of p65, a component of NF-κB (15). DHMEQ has not exhibited evident toxicity in animals (13,15), indicating the tolerance of this compound for NF-κB. In the present study, the anti-lupus property of DHMEQ was investigated in a pristane-induced lupus mouse model. DHMEQ was shown to antagonize the increasing levels of anti-nucleosome, anti-dsDNA and anti-histone autoantibodies, as well as the levels of TNF-α, IL-1β, 6 and 17. In addition, DHMEQ reduced the number of renal lesions caused by pristane, as reflected by milder proteinuria and reduced glomerular pathology. Renal expression levels of p-p38MAPK, p-JNK and NF-κB p65 were significantly downregulated. These results indicate that DHMEQ has a beneficial effect on pristane-induced lupus through regulating the levels of cytokines and the MAPK/JNK/NF-κB signaling pathway.

Elevated constitutive levels of active NF-κB are associated with chronic inflammatory diseases (16). The NF-κB family of transcription factors is regulated by inhibitors, including IκBα. Lower mRNA expression levels of IκBα have been observed in spleens and dendritic cells (DCs) derived from lupus-prone mice, as compared with wild-type mice (6), indicating an abnormal activation of NF-κB in lupus mice. NF-κB can affect the function of DCs and their capacity to regulate adaptive immunity (6). Previous studies have shown that an NF-κB blockade interferes with unwanted T-cell responses, as observed in experimental autoimmune encephalomyelitis (17). In spontaneous lupus model MRL/lpr mice, inhibiting the NF-κB-mediated inflammatory response was shown to be effective for LN (18). The present study has provided new evidence indicating that the pharmacological inhibition of NF-κB in mice with pristane-induced lupus may significantly reduce the effects of lupus disease.

Accumulating evidence has demonstrated the involvement of NF-κB in self-reactive T- and B-lymphocyte development, survival and proliferation, as well as the maintenance of chronic inflammation due to cytokines, including TNF-α, IL-1, 6 and 17 (19). Thus, an NF-κB-mediated inflammatory response may contribute to organ damage in SLE (18,19). In the present study, DHMEQ was found to antagonize the increasing levels of IL-1β, 6 and 17 and TNF-α. In addition, a number of studies indicate that the p38 MAPK/JNK signaling pathway plays an important role in the regulation of cellular and humoral autoimmune responses (20,21). DHMEQ, a specific inhibitor of p38 MAPK, has shown to be effective in an MRL/lpr mouse model of SLE, as demonstrated by improved renal function and the attenuation of histological damage (21). The results of the present study demonstrate that the MAPK/JNK signaling pathway is inhibited by DHMEQ treatment. These results indicate that DHMEQ plays a therapeutic role in SLE by blocking the NF-κB/MAPK/JNK-mediated inflammatory response.

In conclusion, the results of the present study demonstrate that DHMEQ has a beneficial effect on pristane-induced lupus through regulating cytokine levels and the MAPK/JNK/NF-κB signaling pathway. The results support the hypothesis that NF-κB blockade may be an important pharmacological approach for the downmodulation of detrimental autoimmune responses.
